# The effects of ambient temperature on road traffic injuries in Jinan city: a time-stratified case-crossover study based on distributed lag nonlinear model

**DOI:** 10.3389/fpubh.2024.1324191

**Published:** 2024-04-23

**Authors:** YinLu Li, Jie Ren, Wengui Zheng, Jing Dong, Zilong Lu, Zehan Zhang, Aiqiang Xu, Xiaolei Guo, Jie Chu

**Affiliations:** ^1^School of Public Health, Weifang Medical University, Weifang, China; ^2^Department of Non-communicable Disease Control and Prevention, Shandong Center for Disease Control and Prevention, Jinan, China

**Keywords:** ambient temperature, extreme temperature, road traffic injuries, case-crossover study, distributed lag nonlinear model

## Abstract

**Objectives:**

The impact of climate change, especially extreme temperatures, on health outcomes has become a global public health concern. Most previous studies focused on the impact of disease incidence or mortality, whereas much less has been done on road traffic injuries (RTIs). This study aimed to explore the effects of ambient temperature, particularly extreme temperature, on road traffic deaths in Jinan city.

**Methods:**

Daily data on road traffic deaths and meteorological factors were collected among all residents in Jinan city during 2011–2020. We used a time-stratified case-crossover design with distributed lag nonlinear model to evaluate the association between daily mean temperature, especially extreme temperature and road traffic deaths, and its variation in different subgroups of transportation mode, adjusting for meteorological confounders.

**Results:**

A total of 9,794 road traffic deaths were collected in our study. The results showed that extreme temperatures were associated with increased risks of deaths from road traffic injuries and four main subtypes of transportation mode, including walking, Bicycle, Motorcycle and Motor vehicle (except motorcycles), with obviously lag effects. Meanwhile, the negative effects of extreme high temperatures were significantly higher than those of extreme low temperatures. Under low-temperature exposure, the highest cumulative lag effect of 1.355 (95% CI, 1.054, 1.742) for pedal cyclists when cumulated over lag 0 to 6 day, and those for pedestrians, motorcycles and motor vehicle occupants all persisted until 14 days, with ORs of 1.227 (95% CI, 1.102, 1.367), 1.453 (95% CI, 1.214, 1.740) and 1.202 (95% CI, 1.005, 1.438), respectively. Under high-temperature exposure, the highest cumulative lag effect of 3.106 (95% CI, 1.646, 5.861) for motorcycle occupants when cumulated over lag 0 to 12 day, and those for pedestrian, pedal cyclists, and motor vehicle accidents all peaked when persisted until 14 days, with OR values of 1.638 (95% CI, 1.281, 2.094), 2.603 (95% CI, 1.695, 3.997) and 1.603 (95% CI, 1.066, 2.411), respectively.

**Conclusion:**

This study provides evidence that ambient temperature is significantly associated with the risk of road traffic injuries accompanied by obvious lag effect, and the associations differ by the mode of transportation. Our findings help to promote a more comprehensive understanding of the relationship between temperature and road traffic injuries, which can be used to establish appropriate public health policies and targeted interventions.

## 1 Introduction

Road traffic injuries (RTIs) has not only become a social safety problem that cannot be ignored worldwide (1, 2), but a huge challenge to economic development and public health as well (3), with the development of motorization rapidly. The Global Status Report on Road Safety 2018 showed that around 1.35 million people worldwide died from RTIs each year, and 50 million people suffered non-fatal injuries from traffic crashes, some disabled as a result among them (4). RTIs can lead to permanent injuries, such as traumatic brain injury, spinal injury, amputations, which might have devastating, lifelong effects on road users (5, 6). The global economic cost resulted from RTIs is estimated at $1.8 trillion in 2015–2030, equivalent to 0.12 percent of global gross domestic product (7). RTIs are more severe in developing countries than in developed countries, and 93 percent of road traffic deaths occur in low-income and middle-income countries (6).

World Health Organization reported that one-fifth of global road traffic deaths occurred in China (8). RTIs are the leading cause of accident deaths in China and have been on the rise in China since the end of 1980s (9). In recent years, although some studies have reported that RTIs showed a downward trend attributed to road safety laws and various policies in China, the mortality of RTIs is still much higher than that in developed countries (9–11). As a result, it is still a very serious problem in China that should be paid more attention to (2, 12).

In addition to human behaviors, road conditions and vehicles, environmental factors are one of the important reasons leading to RTIs. In recent years, accompanying for the increasing of global warming and air pollution, more and more researches pay attention to the influence of ambient temperature on people’s health. Extreme temperature is currently considered to be an important risk factor for RTIs, which has a direct or indirect impact on the occurring of traffic injuries (13, 14). Weather changes can affect the vehicle itself, road conditions, the judgment and reaction of the driver during the driving process, and the riding environment of the driver and passenger on varying degrees. However, much less studies have focused on the impact on RTIs, especially on RTIs fatalities and the epidemiological evidence for its exact impact is not uniform due to the limitations of traditional research methods (15, 16). To reduce the incidence and mortality of RTIs under extreme temperature conditions, It is urgent need to study the relationship between extreme temperature and RTIs specifically.

A time-stratified case-crossover design approach for distributed lag nonlinear model can be used to study the relationship between environmental factors and health outcomes, overcome multiple biases, and make full use of information from sample data, maximally close to the truth to achieve unbiased estimates. In this study, we used a time-stratified case-crossover design based on distributed lag nonlinear model to examine the relationship between extreme temperature and the risk of RTIs’ deaths, on the basis of 10-year traffic accident statistics from Jinan city, Shandong Province in China. The study will help to develop public health policies and interventions to reduce the negative impacts of climate change on RTIs fatalities in extreme weather conditions.

## 2 Materials and methods

### 2.1 Study area and data collection

Jinan is the capital city of Shandong Province, located at the east coast of China an mid-latitudes. It’s a temperate monsoon climate, characterized by four distinct seasons with warm and rainy summer, cold and dry winter and comfortable spring and autumn transition season.

We collected individual road traffic death records among all inhabitants in Jinan city during 2011–2020 from the Shandong Provincial Death Registration Information Reporting System. Data information were extracted such as date of death, sex, age, and underlying cause of death. The underlying cause of road traffic death was coded by the International Classification of Diseases (version 10), which the coding range was V00-V89. In this study, Road traffic deaths were classified into walking, bicycle, motorcycle, motor vehicle (except motorcycle) and other subtypes according to the mode of transport. Due to the small sample size, deaths caused by injuries in other transportation accidents were excluded from the statistical analysis.

The meteorological data for the same period was obtained from the China Meteorological Data Sharing Service System,^1^ including daily mean temperature (°C), relative humidity (%), wind speed (m/s), and barometric pressure (hPa). To adjust for the potential impacts of air pollutants, daily concentrations of PM2.5 (μg/m^3^), PM10 (μg/m^3^), SO2 (μg/m^3^), NO2 (μg/m3), CO (mg/m^3^), and O3 (μg/m^3^) were extracted from the National Environmental Monitoring Center (NEMC) of China.

### 2.2 Statistical analysis

A time-stratified case-crossover design (15) was used in this study to assess the effects of extreme temperature on RTIs, which is equivalent to a time-stratified self-matched case–control study. Poisson regression was achieved by setting dummy variables (e.g., year, month, day of the week), with the same day of the week of the same month in the same year as the control day (up to 4 control days per case). It can not only control the influence of time trend (such as seasonality and the “day of the week” effect) and meteorological factor, etc., at the same time, the bias caused by individual-level confounders (such as age, intelligence, heredity, etc.) between cases and controls can be avoided, thus the unbiased estimation of parameters can be obtained.

Considering the possible nonlinear relationship between daily mean temperature and RTIs (13, 16, 17), we used conditional Poisson regression with Distributed Lag Non-linear Models (DLNM) to estimate the exposure-response and exposure-lag associations between temperature and RTIs deaths (18–20). The results were reported as odds ratios (OR) and 95% confidence interval (CI). A lag period up to 14 days was used to adequately respond to exposure effects and lag effects. Based on generalized cross-validation (21), we fitted the exposure-response and exposure-lag associations using natural cubic spline with 2 degrees of freedom (df). To avoid potential meteorological and pollutant confounders in the association between ambient temperature and RTIs (22), a natural cubic spline with 3 df was used to control for the effect of relative humidity, and that with 2 df was used to control for pollutants. All data were analyzed using R software (Version 4.2.2), where “gam” and “dlmn” software packages were used to fit the conditional Poisson regression models and exposure-lag-response curves, respectively. The reference temperature, namely minimum-mortality temperature, was determined as the temperature corresponding to the lowest death risk in the exposure-response curve. Extremely low and high temperatures were defined as the 1th and 99th percentile of temperature, respectively.

### 2.3 Sensitivity analyses

We carried out a series of methods to test the robustness of the results. First, the number of lag days in this study was changed from 0–14 days to 0–7 days and 0–21 days so as to test whether a 14-day lagging was sufficient for exposure-response and exposure-lag effects. Second, The df of meteorological confounders were varied from 2 to 4 to check the robustness of the fitted model. Last, the 2.5th or 10th percentile of temperature, defined as extreme low temperature, and the 97.5th or 90th percentile of temperature, defined as extreme high temperature, respectively, were used to fit the temperature-road traffic death lag response curve to check for changes in the model results.

## 3 Result

### 3.1 Descriptive analysis

Table 1 shows the descriptive statistics for daily deaths related to RTIs in Jinan city. A total of 9,794 road traffic injury deaths were collected during 2011–2020 and the daily mean deaths were 3. The percentage of deaths was much higher in male (73.01%), those aged 35–64 years (57.31%), and pedestrians (45.9%) than in female (26.99%), those with other age groups, and those by other transport mode, respectively.

**Table 1:** The descriptive statistics for daily deaths on RTIs among residents with different characteristics in Jinan city, 2011–2020.

		Mean	Minimum	25%	Median	75%	Maximum	Total number
Sex	Male	2	0	1	2	3	9	7,151 (73.01%)
	Female	1	0	0	1	1	6	2,643 (26.99%)
Age	0–34	0	0	0	0	1	5	1810 (18.48%)
	35–64	2	0	1	1	2	9	5,613 (57.31%)
	≥65	1	0	0	0	1	6	2,371 (24.21)
Transport mode	Walking	1	0	0	1	2	9	4,495 (45.9%)
	Bicycle	0	0	0	0	1	4	1,474 (15.05%)
	Motorcycle	0	0	0	0	1	5	1,654 (16.89%)
	Motor vehicle	0	0	0	0	1	8	1,591 (16.24%)
	Other	0	0	0	0	0	3	580 (5.92%)
Total		3	0	1	2	4	12	9,794 (100%)

Table 2 presents the results of descriptive statistics about the daily levels of meteorological indicators and air pollutants. The mean daily mean temperature was 15.13°C, and the daily mean minimum and maximum temperatures were − 12.4°C and 33.8°C, respectively.

**Table 2:** Descriptive statistics about daily level of meteorological and air pollution indicators in Jinan city, 2011–2020.

	Mean	Minimum	25%	Median	75%	Maximum
DMT (°C)	15.1	−12.4	5.7	16.6	24.4	33.8
RH (%)	55	14	40	55	70	100
BP (hPa)	997	975	989	997	1,004	1,022
WS (m/s)	2.4	0.2	1.7	2.2	2.9	8.4
PM2.5 (μg/m^3^)	79	3	42	65	99	443
PM10 (μg/m^3^)	142	5	90	126	174	693
NO2 (μg/m^3^)	48	9	33	44	58	165
CO (mg/m^3^)	1,200	363	811	1,046	1,408	6,555
SO2 (μg/m^3^)	50	5	17	35	62	429
O3-8h (μg/m^3^)	106	5	58	98	148	269

DMT, Daily mean temperature; RH, Relative humidity; BP, Barometric pressure; WS, Wind speed; PM2.5, Particulate matter with aerodynamic diameter less than 2.5 μm; PM10, Particulate matter with aerodynamic diameter less than 10 μm; NO2, Nitrogen dioxide; CO, Carbon monoxide; SO2, Sulfur dioxide; O3-8h, Ozone for 8 h.

Spearman correlation tests were used to identify the correlation between meteorological and air pollution indicators, and the correlation coefficients r were showed in Table 3. *R* ≥ 0.6 was regarded as strong correlation. The daily mean temperature showed a strong correlation with the daily maximum 8-h average ozone and daily atmospheric pressure.

**Table 3:** Correlation coefficients between meteorological and air pollution indicators in Jinan city, 2011–2020.

	DMT	PM2.5	PM10	NO2	SO2	CO	O3-8h	RH	BP	WS
DMT	1	−0.268	−0.240	−0.498	−0.393	−0.404	0.814	0.182	−0.888	0.089
PM2.5		1	0.863	0.648	0.679	0.807	−0.265	0.116	0.212	−0.154
PM10			1	0.674	0.661	0.694	−0.181	−0.149	0.206	−0.036
NO2				1	0.649	0.771	−0.485	−0.078	0.528	−0.347
SO2					1	0.699	−0.364	−0.209	0.342	0.055
CO						1	−0.448	0.188	0.346	−0.317
O3-8h							1	−0.112	−0.694	0.165
RH								1	−0.245	−0.342
BP									1	−0.140
WS										1

All the correlation coefficients were statistically significant with *p* < 0.001.

DMT, Daily mean temperature; RH, Relative humidity; BP, Barometric pressure; WS, Wind speed; PM2.5, Particulate matter with aerodynamic diameter less than 2.5 μm; PM10, Particulate matter with aerodynamic diameter less than 10 μm; NO2, Nitrogen dioxide; CO, Carbon monoxide; SO2, Sulfur dioxide; O3-8h, Ozone for 8 h.

### 3.2 Relationship between ambient temperature and road traffic injuries

**Figure 1:**
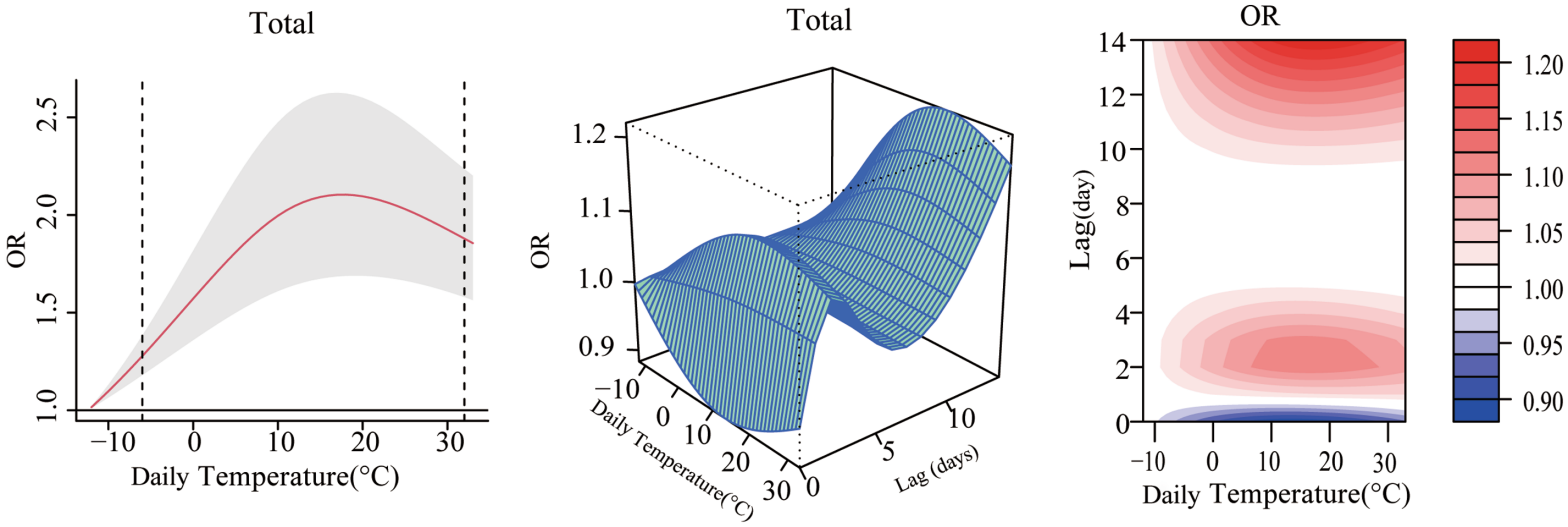
Exposure-response relationship between total RTIs deaths and daily mean temperature in Jinan city, 2011–2020. **(A)** Exposure-response curve. **(B)** 3D effect plot. **(C)** Contour plot.

As shown in Figure 1, the cumulative exposure-response relationship between daily mean temperature and RTIs deaths presented an inverted U-shaped curve. The minimum-mortality temperature (MMT) was −12.4°C when the death risk of RTIs was the lowest. With increasing daily mean temperature, the death risk of RTIs increased gradually, reached the highest at 17.6°C (OR = 2.09, 95% CI: 1.66, 2.63), and then decreased. We found an obviously nonlinear relationship between daily mean temperature and RTIs fatalities from the exposure-lag-response 3D and contour plots. High and low temperatures were found to have “protective” effects on RTIs deaths on the current day. However, with the increase of lag days, both high and low temperature could increase the death risk of RTIs. The death risk reached highest when the lag day was 14th day, and was obviously higher in high temperature than that in low temperature.

**Figure 2:**
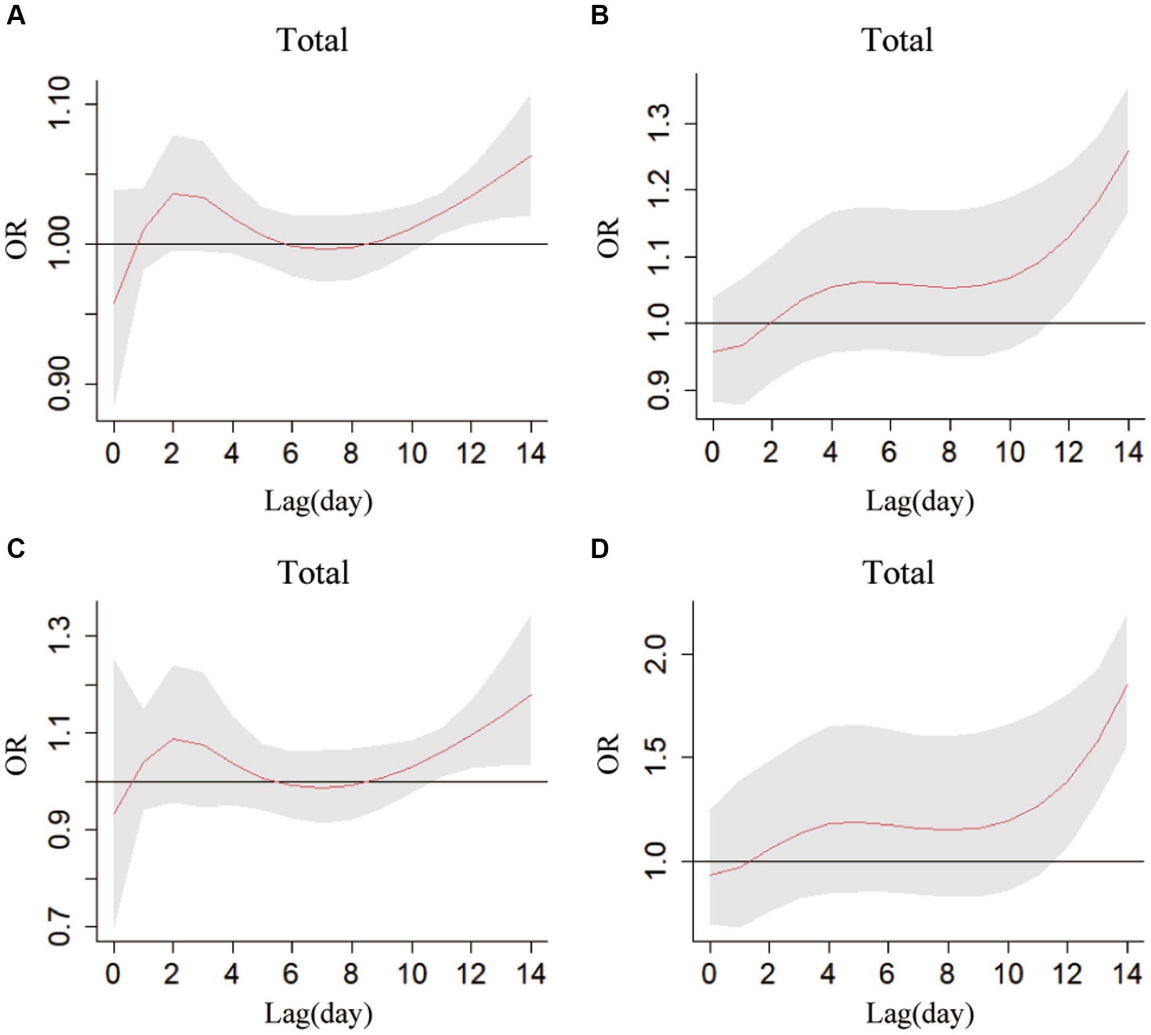
The lagged-response curves for association between Daily Mean Temperature and RTIs fatalities in Jinan City, 2011–2020. **(A)** Single-day lagged low temperature. **(B)** Cumulative lagged low temperature. **(C)** Single-day lagged high-temperature. **(D)** Cumulative lagged high-temperature.

The exposure-lag-response curves were plotted, when the 1st (−6°C) and 99th (32°C) percentile of daily mean temperatures as regarded to the temperature thresholds for extremely low and high temperatures, respectively (Figure 2). The results showed that the effects of extreme low temperature and high temperature on RTIs deaths showed a similar trend with the change of lag time and the death risks of extreme temperature had obviously lag effects on RTIs fatalities. Both extreme high temperature and low temperature were not associated with RTIs deaths on the current day and up to 10 lag days, while the association appeared until 11th lag day, and then cumulatively reached highest over lag days of 14. The effect of extreme high temperature on RTIs fatalities was obviously higher than that of extreme low temperature, no matter single-day effect or cumulative effect.

**Figure 3:**
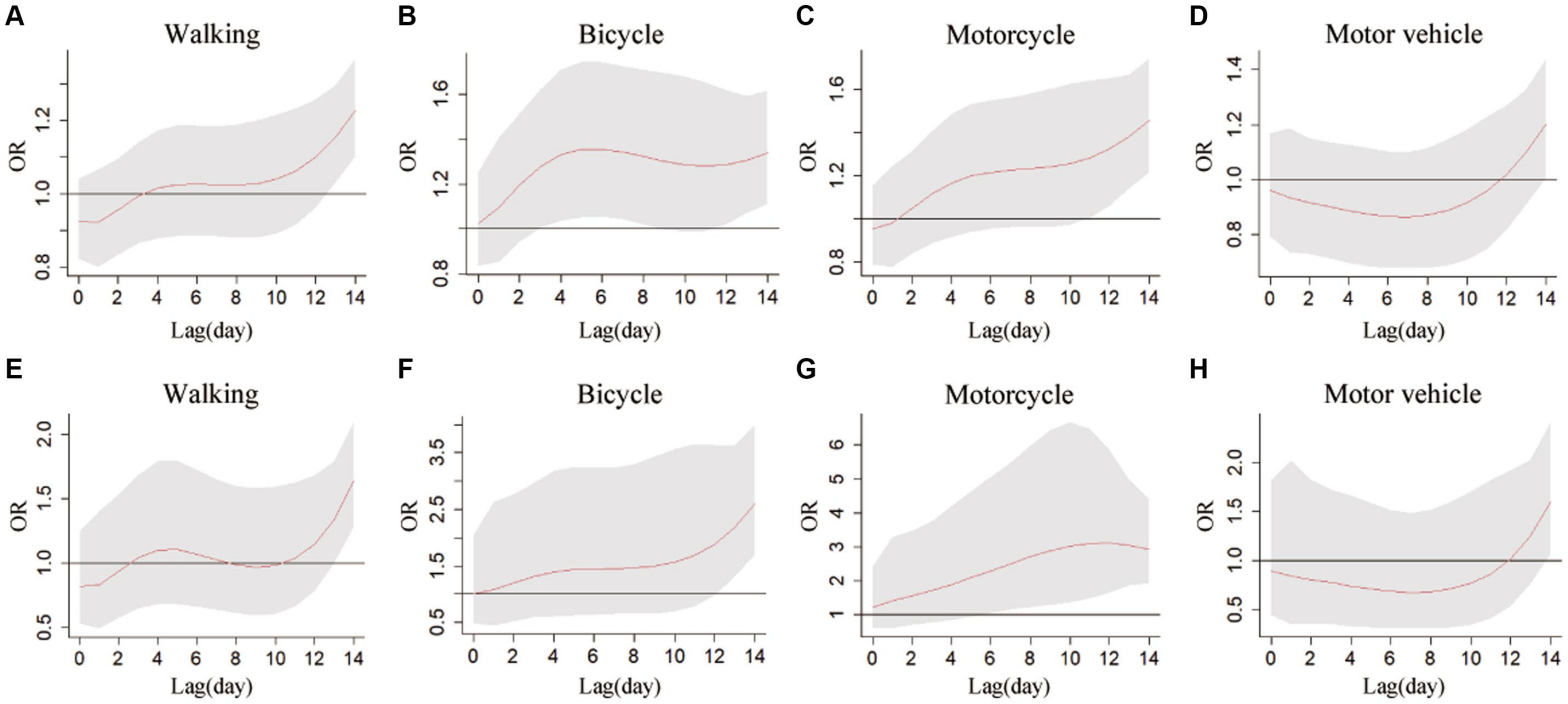
Cumulative Effects of extreme low-temperature and high-temperature on RTIs fatalities over lag days 0–14 in different subtypes of transport mode, in Jinan city, 2011–2020. **(A)** Cumulative effect at extreme low temperature (−6°C) on pedestrian injuries in transport accidents. **(B)** Cumulative effect at extreme low temperature (−6°C) on cyclist injuries in transport accidents. **(C)** Cumulative effect at extreme low temperature (−6°C) on motorcyclist injuries. **(D)** Cumulative effect at extreme low temperature (−6°C) on motor vehicle (except motorcycles) occupant injuries in transport accidents. **(E)** Cumulative effect at extreme high temperature (32°C) on pedestrian injuries in transport accidents. **(F)** Cumulative effect at extreme high temperature (32°C) in cyclist injuries in transport accidents. **(G)** Cumulative effect at extreme high temperature (32°C) on motorcyclist injuries in transport accidents. **(H)** Cumulative effect at extreme high temperature (32°C) on motor vehicle (except motorcycles) occupant injuries in transport accidents.

The results have shown that exposure to low temperature has a significant lag effect on the risk of death from different types of RTIs. When exposed to low temperature, the cumulative effect of road injury mortality risk for pedestrians and motorcycle riders was significant on Lag11 and Lag13, respectively, and the maximum single-day effect was appeared on Lag14, with OR values of 1.227 (95% CI: 1102, 1.367) and 1.453 (95% CI: 1.214, 1.74). The risk of death for cyclists was occurred on Lag3 and reached the maximum on Lag6 (OR = 1.355, 95% CI: 1.054, 1.742), disappeared after 6 days, then reappeared on Lag12 and reached the maximum on Lag14. The mortality risk for passengers of motor vehicles was statistically significant on Lag14 (OR = 1.202, 95% CI: 1.005, 1.438). Exposure to high temperatures also has a lag effect on the risk of RTIs death among residents. The cumulative effect of high temperature on the risk of death for pedestrians, cyclists, and motor vehicle passengers all reached the maximum on Lag14, with OR values of 1.638 (95% CI: 1.281, 2.094), 2.603 (95% CI: 1.695, 3.997), and 1.603 (95% CI: 1.066, 2.411), respectively. The mortality risk for motorcycle riders increased significantly on Lag6, reaching a maximum OR of 3.106 (1.646, 5.861) on Lag12 (Figure 3; Table 4).

**Table 4:** Cumulative effect estimates of extreme low temperature and high temperature related to RTIs fatalities over lag days 0–14 by different mode of transportation.

	Walking OR (95% CI)	Bicycle OR (95% CI)	Motorcycle OR (95% CI)	Motor vehicle OR (95% CI)
Extreme low temperature (−6°C)				
Lag01	0.924 (0.800, 1.066)	1.097 (0.855, 1.408)	0.981 (0.774, 1.243)	0.935 (0.736, 1.188)
Lag02	0.957 (0.834, 1.097)	1.193 (0.941, 1.511)	1.047 (0.835, 1.313)	0.917 (0.730, 1.152)
Lag03	0.993 (0.864, 1.140)	**1.277 (1.005, 1.623)**	1.116 (0.886, 1.405)	0.901 (0.715, 1.135)
Lag04	1.016 (0.879, 1.174)	**1.331 (1.037, 1.709)**	1.166 (0.916, 1.485)	0.886 (0.696, 1.128)
Lag05	1.025 (0.885, 1.186)	**1.354 (1.052, 1.744)**	1.197 (0.937, 1.529)	0.873 (0.684, 1.114)
Lag06	1.026 (0.887, 1.187)	**1.355 (1.054, 1.742)**	1.215 (0.953, 1.549)	0.865 (0.679, 1.102)
Lag07	1.024 (0.885, 1.185)	**1.341 (1.043, 1.725)**	1.224 (0.959, 1.562)	0.863 (0.677, 1.101)
Lag08	1.024 (0.882, 1.189)	**1.321 (1.021, 1.710)**	1.231 (0.960, 1.579)	0.870 (0.678, 1.116)
Lag09	1.028 (0.881, 1.199)	1.301 (0.998, 1.696)	1.240 (0.960, 1.602)	0.887 (0.686, 1.146)
Lag010	1.040 (0.891, 1.214)	1.286 (0.985, 1.679)	1.256 (0.971, 1.624)	0.915 (0.707, 1.185)
Lag011	1.063 (0.917, 1.234)	1.28 0(0.990, 1.653)	**1.282 (1.002, 1.640)**	0.958 (0.748, 1.227)
Lag012	1.101 (0.963, 1.258)	**1.285 (1.02, 1.619)**	**1.322 (1.059, 1.650)**	1.018 (0.815, 1.271)
Lag013	**1.154 (1.029, 1.294)**	**1.304 (1.07, 1.591)**	**1.378 (1.139, 1.666)**	1.098 (0.908, 1.327)
Lag014	**1.227 (1.102, 1.367)**	**1.34 (1.111, 1.615)**	**1.453 (1.214, 1.740)**	**1.202 (1.005, 1.438)**
Extreme high temperature (33°C)				
Lag01	0.833 (0.493, 1.408)	1.079 (0.442, 2.633)	1.397 (0.595, 3.283)	0.838 (0.348, 2.020)
Lag02	0.939 (0.574, 1.535)	1.204 (0.522, 2.773)	1.552 (0.696, 3.463)	0.804 (0.353, 1.833)
Lag03	1.049 (0.65, 1.691)	1.319 (0.586, 2.968)	1.710 (0.780, 3.748)	0.773 (0.347, 1.721)
Lag04	1.105 (0.68, 1.795)	1.393 (0.611, 3.176)	1.888 (0.849, 4.203)	0.739 (0.327, 1.667)
Lag05	1.105 (0.681, 1.795)	1.427 (0.626, 3.253)	2.087 (0.938, 4.645)	0.707 (0.314, 1.592)
Lag06	1.073 (0.665, 1.729)	1.439 (0.639, 3.241)	**2.298 (1.045, 5.054)**	0.683 (0.307, 1.519)
Lag07	1.029 (0.640, 1.654)	1.445 (0.644, 3.240)	**2.510 (1.148, 5.492)**	0.673 (0.304, 1.488)
Lag08	0.991 (0.613, 1.603)	1.459 (0.644, 3.305)	**2.711 (1.229, 5.981)**	0.679 (0.304, 1.516)
Lag09	0.973 (0.598, 1.585)	1.495 (0.652, 3.431)	**2.884 (1.293, 6.432)**	0.707 (0.313, 1.598)
Lag010	0.985 (0.608, 1.596)	1.567 (0.689, 3.565)	**3.016 (1.365, 6.662)**	0.764 (0.342, 1.710)
Lag011	1.038 (0.662, 1.629)	1.689 (0.783, 3.647)	**3.093 (1.474, 6.488)**	0.860 (0.406, 1.824)
Lag012	1.147 (0.780, 1.685)	1.882 (0.972, 3.645)	**3.106 (1.646, 5.861)**	1.011 (0.533, 1.919)
Lag013	1.334 (0.993, 1.791)	**2.173 (1.304, 3.620)**	**3.051 (1.87, 4.978)**	1.244 (0.764, 2.026)
Lag014	**1.638 (1.281, 2.094)**	**2.603 (1.695, 3.997)**	**2.930 (1.939.4.429)**	**1.603 (1.066, 2.411)**

The bold values means statistically significant results.

### 3.3 Results of sensitivity analysis

The results of the sensitivity analysis were robust, as described in Supplementary material. The exposure-response curves and relative risks were similar when changing the maximum lag days from 14 to 7 and 21, respectively (Supplementary Figure S1). The effects of confounders with different df on RTIs remained unchanged when exposed to extreme low and high temperature, respectively, over lag day 0–14 (Supplementary Table S1). The associations between extreme low and high temperature and RTIs fatalities were stable over lag days 0–14 using different threshold values of extreme temperature (Supplementary Figure S2).

## 4 Discussion

In this study, we examined the association between ambient temperature, particularly extreme temperatures, and road injury deaths in Jinan city. The results confirmed that extreme high and low temperatures were positively associated with the risk of RTIs fatalities with a significant lag effect. Meanwhile, the effect of extreme high temperature on RTIs fatalities was significantly higher than that of extreme low temperatures. In addition, the effects of extreme high temperature on RTIs fatalities were obviously stronger among cyclist and motorcyclist than those among pedestrians and motorized vehicle (except motorcycles) personnel.

Our study provided evidence for the association between extreme temperatures and road accident deaths in Jinan city, Shandong Province. The present study found an inverted U-shaped curve in the relationship between daily mean temperature and RTIs fatalities, with the highest risk occurring at moderate temperatures (17.6°C). Similar to our findings, a study in Beijing (China) found that accidental injuries and deaths occurred more frequently on warm days, with the highest likelihood of emergency treatment for accidental injuries occurring at 26°C instead of extreme temperatures (23). An Italian study also found that the peak of workplace accidents occurred at hot but not extreme temperatures (24). The increased risk of RTIs in warm temperatures may be related to the increased frequency with which people go out or traveling (25). With the rapid development of road transportation and urban service industry, increased traffic jams and poor self-protection of non-motor vehicles make road traffic injuries more likely to occur (26).

Previous study have shown that high and low temperatures were obviously correlated with RTIs, and the effect of extreme hot weather was significantly higher than that of extreme cold weather (27), which was consistent with the results of this paper on the impact of high temperatures. Wu et al. (16) showed that there was a significant positive correlation between fatal traffic accidents and heat waves in the United States. Bergel-Hayat et al. (28) believed that small temperature changes will have a significant impact on the risk of traffic accidents. For every 1°C increase in monthly mean temperature, the number of collisions increases by 1–2%. These studies all suggested an association between ambient temperature and RTIs. In a study of meta-analysis using daily mean temperatures, higher temperatures was found to increased the risk of traffic injuries by 2.4% (RR = 1.024, 95% CI 0.939, 1.116) (29). Basagaña et al. (30) found that for every 1°C increase in maximum temperature, there was a significant increase in the estimated risk (OR = 1.1, 95% CI: 0.1, 2.1) of crashes due to the driver performance factor. The susceptibility to crashes exposing to high-temperature was related to multiple mechanisms, such as human behavior, vehicle conditions, and environmental factors (31). Higher temperatures may lead to reduced vigilance and inattention which directly resulted in the poor driving behaviors (32–35). At the same time, high temperatures may increase the likelihood of dangerous behaviors such as running red lights and driving in the wrong lane (36). In addition, changes in ambient temperatures may also result in the occurrence of disease, which increased the risk of traffic accidents for road users when they traveled (37). These reasons ultimately lead to an increase in the risk of road collisions in high-temperature environments.

Our study found that, pedestrians, cyclists and motorcyclists are at greater risk of injury than motorized vehicle (except motorcycles) personnel, regardless of exposure to low temperature or high temperature, which is in line with the results of the World Health Organization report on vulnerable groups of road traffic injuries. In our study, pedestrians, bicyclists and motorcyclists were the vulnerable groups of road traffic injuries, accounting for more than half of all RTIs deaths (77.84%). It may be related to their occupations and the modes of transport they used. For example, with the rapid development of takeaway, express delivery industry, bike-sharing and other industries, the use of bicycles and motorcycles has been increasing. Road crashes and injuries are more likely to occur resulted from increased traffic jams and the poor self-protection performance of road users in above travel modes (38). In addition, ambient temperature has a greater effect on two-wheeler (bicycle and motorcycle) users than four-wheeler vehicle users due to their direct exposure to the external environment. A study in Belgium has shown an increase in the frequency of road traffic accidents among two-wheeled vehicle (bicycle and motorcycle) users in warm weather (39). Daanen et al. (40) noted that in extremely cold environments, the driving status of motorcyclists become worse due to the cold, thus increasing the risk of traffic accidents. Similarly, studies have shown that motorcyclists will distract their attention because of coping with thermal stress under high temperature exposure, and then indirectly reduce their ability to cope with various traffic conditions (41). Moreover, the temperature may affect resident’s choice of travel mode so as to make more appropriate travel decisions. Gan found that the effect of temperature on the number of bicycle trips was significant. Compared with spring, the number of bicycle trips increased in autumn, but decreased in summer and winter (42). Hu thought that residents in cold areas could reduce the use of bicycles and electric vehicles, and more use of motor vehicles to travel (43).

There are inconsistent evidences regarding to the lag effects of ambient temperature on RTIs. Some studies suggested that the risk of traffic injuries was significantly associated with high and low temperatures, with significant lag effects, which was consistent with the findings in our study (17, 27). A study in Dalian, China, using distributed lag nonlinear model found that both high (RR = 1.198, 95% CI: 1.017–1.411) and low temperatures (RR = 1.017, 95% CI: 1.001–1.035) increased the risk of RTIs, with a cumulative lagged effect that beyond day 7 (44). This indicates that the effects of ambient temperature on human health may continue to affect road users for several days, resulting in a lagged effect of ambient temperature on traffic injuries. The mechanism of the lagged response between temperature and road traffic accidents is not completely clear. Some scholars believed high temperatures can result in prolonged heat stress and sleep disturbances to increase the risk of daytime fatigue driving, and ultimately lead to road traffic accidents (45). Ma et al. (23) pointed out that extreme temperature leaded to the changes in the body ‘s immune system and body temperature system, and the intensity of its regulation indirectly affected the state of road users over a period of time. In addition, high temperatures may lead to 5-hydroxytryptamine dysfunction (46) and brain damage (47), which will have a negative impact on people’s decision-making ability in the long term and increase the risk of road traffic accidents. However, some other studies have shown that there is no or less lag in the effect of temperature on RTIs. Lee et al. showed that the effect of high temperature on RTIs reached the maximum on the same day, while the effect of low temperature was significant with 2-days lag (14). The inconsistence in research findings may be due to differences in the definition of injury types, subgroups of the target population, and meteorological and geographical conditions. Focusing on the lag effect will help us better understand the impacts of environmental temperature, especially extreme temperature, on RTIs, so as to effectively control and prevent temperature-related traffic injuries.

There are some limitations in this study. First, there may be some error in the measurements of temperature at the time of death from road accidents because the measurements of daily temperature were collected from fixed monitoring sites in Jinan city, and therefore may not have been the actual temperature of the location where the deceased patient was exposed. Second, we did not distinguish ally the road traffic injuries related to occupations in our study. Given that most previous studies on unintentional injuries have focused on occupational injuries, direct comparisons may have had some impact on the results. Third, we did not consider the impact on lag effects of high and low temperatures caused by the possible intervention of early warning systems for heat or cold waves. In addition, the impact of rainfall on traffic accidents is evident, however, due to the unavailability of data, this study did not consider the confounding effects of rainfall on temperature. And last, The results may be given rise to some bias in subgroup analysis due to the small sample sizes for each subgroup of road traffic injuries. In the future, more large-scale and meticulous studies are needed to determine the correlation between temperature and road traffic injuries.

## 5 Conclusion

In summary, extreme low and high temperature were positively associated with the increased risk of road traffic fatalities in Jinan city, with a significant lag effect, and the effect of extreme high temperature on RTIs fatalities was significantly higher than that of extreme low temperatures. The risk association and days of lag effects were different between extreme temperatures and RTIs fatalities in subtgroups of transportation mode. This study helps to develop public health policies and interventions to reduce the negative impacts of climate change on road traffic injuries in extreme weather conditions.

## Data availability statement

The raw data supporting the conclusions of this article will be made available by the authors, without undue reservation.

## Author contributions

YL: Writing – original draft, Writing – review & editing, Data curation, Formal analysis, Methodology, Visualization, Software. JR: Writing – review & editing, Project administration. WZ: Writing – review & editing, Supervision. JD: Writing – review & editing, Project administration. ZL: Writing – review & editing, Data curation, Formal analysis. ZZ: Writing – review & editing, Data curation. XG: Writing – review & editing, Conceptualization, Data curation, Funding acquisition, Project administration, Validation. JC: Writing – review & editing, Conceptualization, Data curation, Funding acquisition, Project administration, Validation. AX: Writing – review & editing, Conceptualization, Funding acquisition, Project administration, Validation.

## Footnotes

^1^ http://data.cma.cn/

## References

[1] Rosen HE Bari I Paichadze N Peden M Khayesi M Monclús J. Global road safety 2010-18: an analysis of global status reports. Injury. (2022). doi: 10.1016/j.injury.2022.07.030, PMID: 35906119

[2] Qi M Hu X Li X Wang X Shi X. Analysis of road traffic injuries and casualties in China: a ten-year nationwide longitudinal study. PeerJ. (2022) 10:e14046. doi: 10.7717/peerj.14046, PMID: 36128192 PMC9482767

[3] Ahmed SK Mohammed MG Abdulqadir SO El-Kader RGA El-Shall NA Chandran D. Road traffic accidental injuries and deaths: a neglected global health issue. Health Sci Rep. (2023) 6:e1240. doi: 10.1002/hsr2.1240, PMID: 37152220 PMC10154805

[4] Li X Ma Q Wang W Wang B. Influence of weather conditions on the intercity travel mode choice: a case of Xi'an. Comput Intell Neurosci. (2021) 2021:1–15. doi: 10.1155/2021/9969322 PMC8407973 34475950

[5] Bakhsh A Aljuzair AH Eldawoody H. An epidemiological overview of spinal trauma in the Kingdom of Saudi Arabia. Spine Surg Related Res. (2020) 4:300–4. doi: 10.22603/ssrr.2019-0118, PMID: 33195853 PMC7661028

[6] Maas AIR Menon DK Manley GT Abrams M Åkerlund C Andelic N. Traumatic brain injury: progress and challenges in prevention, clinical care, and research. Lancet Neurol. (2022) 21:1004–60. doi: 10.1016/S1474-4422(22)00309-X, PMID: 36183712 PMC10427240

[7] Bezabih Y Tesfaye B Melaku B Asmare H. Pattern of orthopedic injuries related to road traffic accidents among patients managed at the emergency Department in Black Lion Hospital, Addis Ababa, Ethiopia, 2021. Open Access Emerg Med. (2022) 14:347–54. doi: 10.2147/OAEM.S368324, PMID: 35903799 PMC9314752

[8] Liu G Chen S Zeng Z Cui H Fang Y Gu D. Risk factors for extremely serious road accidents: results from national road accident statistical annual report of China. PLoS One. (2018) 13:e0201587. doi: 10.1371/journal.pone.0201587, PMID: 30067799 PMC6070265

[9] Wang SY Li YH Chi GB Xiao SY Ozanne-Smith J Stevenson M. Injury-related fatalities in China: an under-recognised public-health problem. Lancet (London, England). (2008) 372:1765–73. doi: 10.1016/S0140-6736(08)61367-7 18930527

[10] Ren K Miao L Lyu J. The temporal trend of road traffic mortality in China from 2004 to 2020. SSM Popul Health. (2023) 24:101527. doi: 10.1016/j.ssmph.2023.101527, PMID: 37885752 PMC10597791

[11] Chu J Xu ML Lu ZL Liu J Chen XX Dong J. Mortality level and tendency of road traffic injury in Shandong Province from 2012 to 2020. Zhonghua Yu Fang Yi Xue Za Zhi. (2022) 56:1307–13. doi: 10.3760/cma.j.cn112150-20220520-00510, PMID: 36207896

[12] Yuan P Qi G Hu X Qi M Zhou Y Shi X. Characteristics, likelihood and challenges of road traffic injuries in China before COVID-19 and in the postpandemic era. Humanit Soc Sci Commun. (2023) 10:2. doi: 10.1057/s41599-022-01482-0, PMID: 36619597 PMC9808728

[13] Zhan ZY Yu YM Chen TT Xu LJ Ou CQ. Effects of hourly precipitation and temperature on road traffic casualties in Shenzhen, China (2010-2016): a time-stratified case-crossover study. Sci Total Environ. (2020) 720:137482. doi: 10.1016/j.scitotenv.2020.137482, PMID: 32145618

[14] Lee H Myung W Kim H Lee EM Kim H. Association between ambient temperature and injury by intentions and mechanisms: a case-crossover design with a distributed lag nonlinear model. Sci Total Environ. (2020) 746:141261. doi: 10.1016/j.scitotenv.2020.141261, PMID: 32745866

[15] Wu Y Li S Guo Y. Space-time-stratified case-crossover Design in Environmental Epidemiology Study. Health Data Sci. (2021) 2021:9870798. doi: 10.34133/2021/9870798 38487511 PMC10880144

[16] Wu CYH Zaitchik BF Gohlke JM. Heat waves and fatal traffic crashes in the continental United States. Accid Anal Prev. (2018) 119:195–201. doi: 10.1016/j.aap.2018.07.025, PMID: 30048841 PMC6675573

[17] Gariazzo C Bruzzone S Finardi S Scortichini M Veronico L Marinaccio A. Association between extreme ambient temperatures and general indistinct and work-related road crashes. A nationwide study in Italy. Accid Anal Prev. (2021) 155:106110. doi: 10.1016/j.aap.2021.106110, PMID: 33836417

[18] Gasparrini A. Distributed lag linear and non-linear models in R: the package dlnm. J Stat Softw. (2011) 43:1–20. doi: 10.18637/jss.v043.i08 PMC3191524 22003319

[19] Ai H Nie R Wang X. Evaluation of the effects of meteorological factors on COVID-19 prevalence by the distributed lag nonlinear model. J Transl Med. (2022) 20:170. doi: 10.1186/s12967-022-03371-1, PMID: 35410263 PMC8995909

[20] Gasparrini A Armstrong B. Reducing and meta-analysing estimates from distributed lag non-linear models. BMC Med Res Methodol. (2013) 13:1. doi: 10.1186/1471-2288-13-1, PMID: 23297754 PMC3599933

[21] Kucheryavskiy S Rodionova O Pomerantsev A. Procrustes cross-validation of multivariate regression models. Anal Chim Acta. (2023) 1255:341096. doi: 10.1016/j.aca.2023.341096 37032062

[22] Guo Y Punnasiri K Tong S. Effects of temperature on mortality in Chiang Mai city, Thailand: a time series study. Environ Health. (2012) 11:36. doi: 10.1186/1476-069X-11-36 22613086 PMC3391976

[23] Ma P Wang S Fan X Li T. The impacts of air temperature on accidental casualties in Beijing, China. Int J Environ Res Public Health. (2016) 13:1073. doi: 10.3390/ijerph13111073, PMID: 27827842 PMC5129283

[24] Morabito M Cecchi L Crisci A Modesti PA Orlandini S. Relationship between work-related accidents and hot weather conditions in Tuscany (Central Italy). Ind Health. (2006) 44:458–64. doi: 10.2486/indhealth.44.458, PMID: 16922190

[25] He L Liu C Shan X Zhang L Zheng L Yu Y. Impact of high temperature on road injury mortality in a changing climate, 1990-2019: a global analysis. Sci Total Environ. (2023) 857:159369. doi: 10.1016/j.scitotenv.2022.159369, PMID: 36228793

[26] Ma C Yang D Zhou J Feng Z Yuan Q. Risk riding behaviors of urban E-bikes: a literature review. Int J Environ Res Public Health. (2019) 16:2308. doi: 10.3390/ijerph16132308, PMID: 31261838 PMC6651001

[27] Zare Sakhvidi MJ Yang J Mohammadi D FallahZadeh H Mehrparvar A Stevenson M. Extreme environmental temperatures and motorcycle crashes: a time-series analysis. Environ Sci Pollut Res Int. (2022) 29:76251–62. doi: 10.1007/s11356-022-21151-8, PMID: 35668256 PMC9553821

[28] Bergel-Hayat R Debbarh M Antoniou C Yannis G. Explaining the road accident risk: weather effects. Accid Anal Prev. (2013) 60:456–65. doi: 10.1016/j.aap.2013.03.006, PMID: 23928504

[29] Liang M Min M Guo X Song Q Wang H Li N. The relationship between ambient temperatures and road traffic injuries: a systematic review and meta-analysis. Environ Sci Pollut Res Int. (2022) 29:50647–60. doi: 10.1007/s11356-022-19437-y, PMID: 35235122

[30] Basagaña X Escalera-Antezana JP Dadvand P Llatje Ò Barrera-Gómez J Cunillera J. High ambient temperatures and risk of motor vehicle crashes in Catalonia, Spain (2000-2011): a time-series analysis. Environ Health Perspect. (2015) 123:1309–16. doi: 10.1289/ehp.1409223, PMID: 26046727 PMC4671248

[31] Theofilatos A Yannis G. A review of the effect of traffic and weather characteristics on road safety. Accid Anal Prev. (2014) 72:244–56. doi: 10.1016/j.aap.2014.06.017, PMID: 25086442

[32] Abdel-Aty M Ekram AA Huang H Choi K. A study on crashes related to visibility obstruction due to fog and smoke. Accid Anal Prev. (2011) 43:1730–7. doi: 10.1016/j.aap.2011.04.003 21658500

[33] Abedi L Sadeghi-Bazargani H. Epidemiological patterns and risk factors of motorcycle injuries in Iran and eastern Mediterranean region countries: a systematic review. Int J Inj Control Saf Promot. (2017) 24:263–70. doi: 10.1080/17457300.2015.1080729, PMID: 26394286

[34] Hammad HM Ashraf M Abbas F Bakhat HF Qaisrani SA Mubeen M. Environmental factors affecting the frequency of road traffic accidents: a case study of sub-urban area of Pakistan. Environ Sci Pollut Res Int. (2019) 26:11674–85. doi: 10.1007/s11356-019-04752-8 30888616

[35] Nazif-Munoz JI Martínez P Williams A Spengler J. The risks of warm nights and wet days in the context of climate change: assessing road safety outcomes in Boston, USA and Santo Domingo, Dominican Republic. Inj Epidemiol. (2021) 8:47. doi: 10.1186/s40621-021-00342-w, PMID: 34281624 PMC8287725

[36] Fu C Liu H. Investigating influence factors of traffic violations at signalized intersections using data gathered from traffic enforcement camera. PLoS One. (2020) 15:e0229653. doi: 10.1371/journal.pone.0229653, PMID: 32130254 PMC7055877

[37] Luo J He G Xu Y Chen Z Xu X Peng J. The relationship between ambient temperature and fasting plasma glucose, temperature-adjusted type 2 diabetes prevalence and control rate: a series of cross-sectional studies in Guangdong Province, China. BMC Public Health. (2021) 21:1534. doi: 10.1186/s12889-021-11563-5, PMID: 34380442 PMC8356456

[38] Hou K Zhang L Xu X Yang F Chen B Hu W. Ambient temperatures associated with increased risk of motor vehicle crashes in New York and Chicago. Sci Total Environ. (2022) 830:154731. doi: 10.1016/j.scitotenv.2022.154731, PMID: 35331770

[39] Masterson JM Richardson FA. Humidex: a method of quantifying human discomfort due to excessive heat and humidity. Downsview: Environment Canada (1979).

[40] Daanen HA van de Vliert E Huang X. Driving performance in cold, warm, and thermoneutral environments. Appl Ergon. (2003) 34:597–602. doi: 10.1016/S0003-6870(03)00055-3, PMID: 14559420

[41] Hancock PA Ross JM Szalma JL. A meta-analysis of performance response under thermal stressors. Hum Factors. (2007) 49:851–77. doi: 10.1518/001872007X230226 17915603

[42] Sung H. Causal impacts of the COVID-19 pandemic on daily ridership of public bicycle sharing in Seoul. Sustain Cities Soc. (2023) 89:104344. doi: 10.1016/j.scs.2022.104344, PMID: 36514674 PMC9731812

[43] ZQ H. Research on the influencing factors of winter travel mode choice for residents in cold regions. Lanzhou Gansu Province: Lanzhou Jiaotong University. (2023).

[44] Liang M Zhao D Wu Y Ye P Wang Y Yao Z. Short-term effects of ambient temperature and road traffic accident injuries in Dalian, northern China: a distributed lag non-linear analysis. Accid Anal Prev. (2021) 153:106057. doi: 10.1016/j.aap.2021.106057, PMID: 33647596

[45] Zheng G Li K Wang Y. The effects of high-temperature weather on human sleep quality and appetite. Int J Environ Res Public Health. (2019) 16:270. doi: 10.3390/ijerph16020270, PMID: 30669302 PMC6351950

[46] Brewerton TD Putnam KT Lewine RRJ Risch SC. Seasonality of cerebrospinal fluid monoamine metabolite concentrations and their associations with meteorological variables in humans. J Psychiatr Res. (2018) 99:76–82. doi: 10.1016/j.jpsychires.2018.01.004, PMID: 29427844 PMC5849528

[47] Bazille C Megarbane B Bensimhon D Lavergne-Slove A Baglin AC Loirat P. Brain damage after heat stroke. J Neuropathol Exp Neurol. (2005) 64:970–5. doi: 10.1097/01.jnen.0000186924.88333.0d 16254491

