# Supplementary material for: The effects of ambient temperature on road traffic injuries in Jinan city: a time-stratified case-crossover study based on distributed lag nonlinear model
Source: Front Public Health. 2024 Apr 23;12:1324191. doi: 10.3389/fpubh.2024.1324191 (PMC11074458; doi:10.3389/fpubh.2024.1324191)
Supplement: Supplementary file 1 [file Data_Sheet_1.docx]

**Abbreviation**

| Abbreviation | The full name in English |
| --- | --- |
| df | Degree of freedom |
| RTIs | Road traffic injuries |
| OR | Odds ratio |
| CI | Confidence interval |
