# Supplementary material for: The effects of ambient temperature on road traffic injuries in Jinan city: a time-stratified case-crossover study based on distributed lag nonlinear model
Source: Front Public Health. 2024 Apr 23;12:1324191. doi: 10.3389/fpubh.2024.1324191 (PMC11074458; doi:10.3389/fpubh.2024.1324191)
Supplement: Supplementary file 2 [file Data_Sheet_2.docx]

Supplementary Material

## Supplementary Figures

**
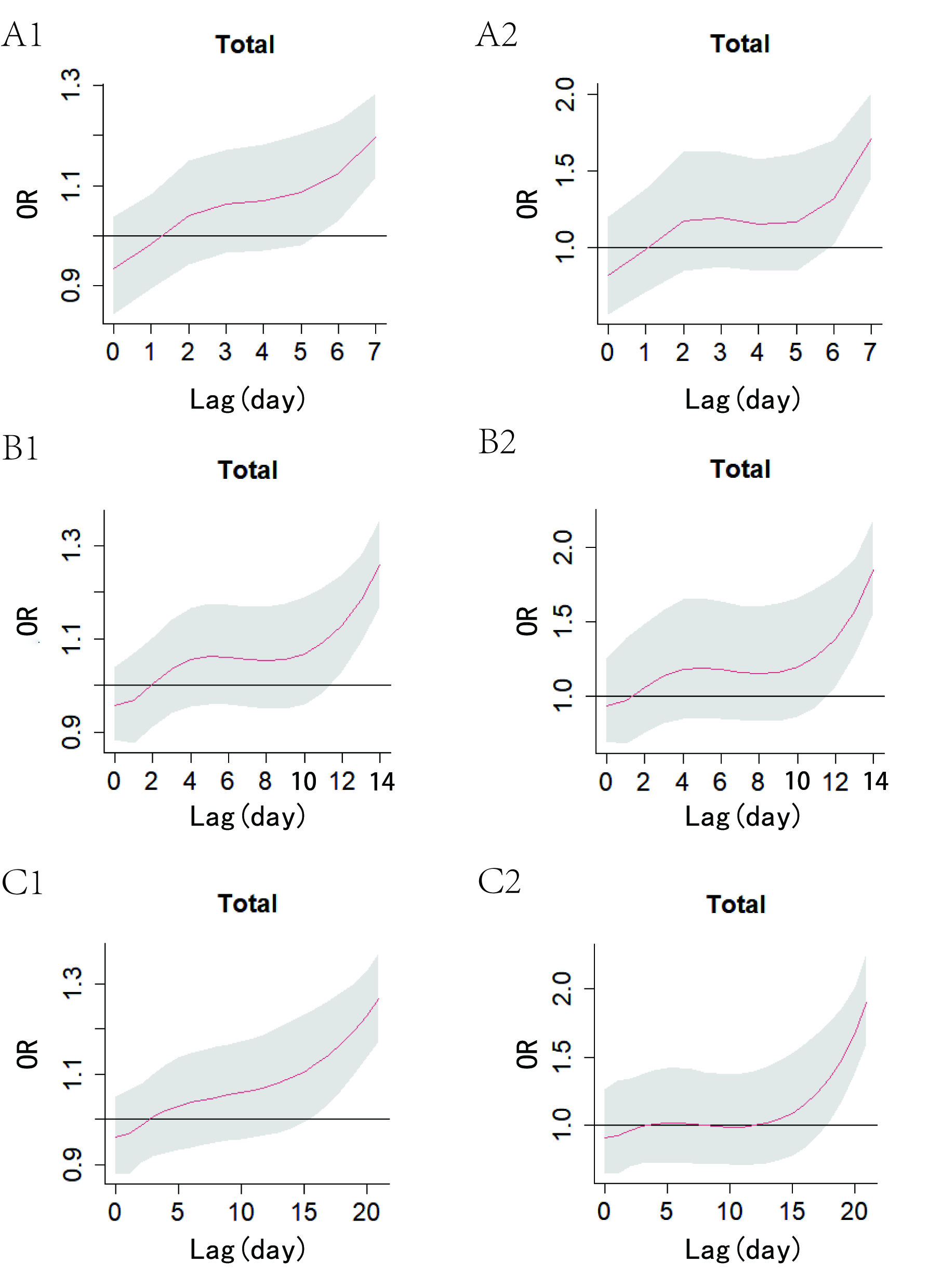
**

**Figure S1**. Cumulative lag effects of extreme low and high temperature on total mortality of RTIs over lag days 0-7, 0-14, 0-21 (A1, A2: ORs of extreme low and high temperature, over lag days 0-7; B1, B2 ORs of extreme low and high temperature, over lag days 0-14; C1, C2: ORs of extreme low and high temperature, over lag days 0-21)


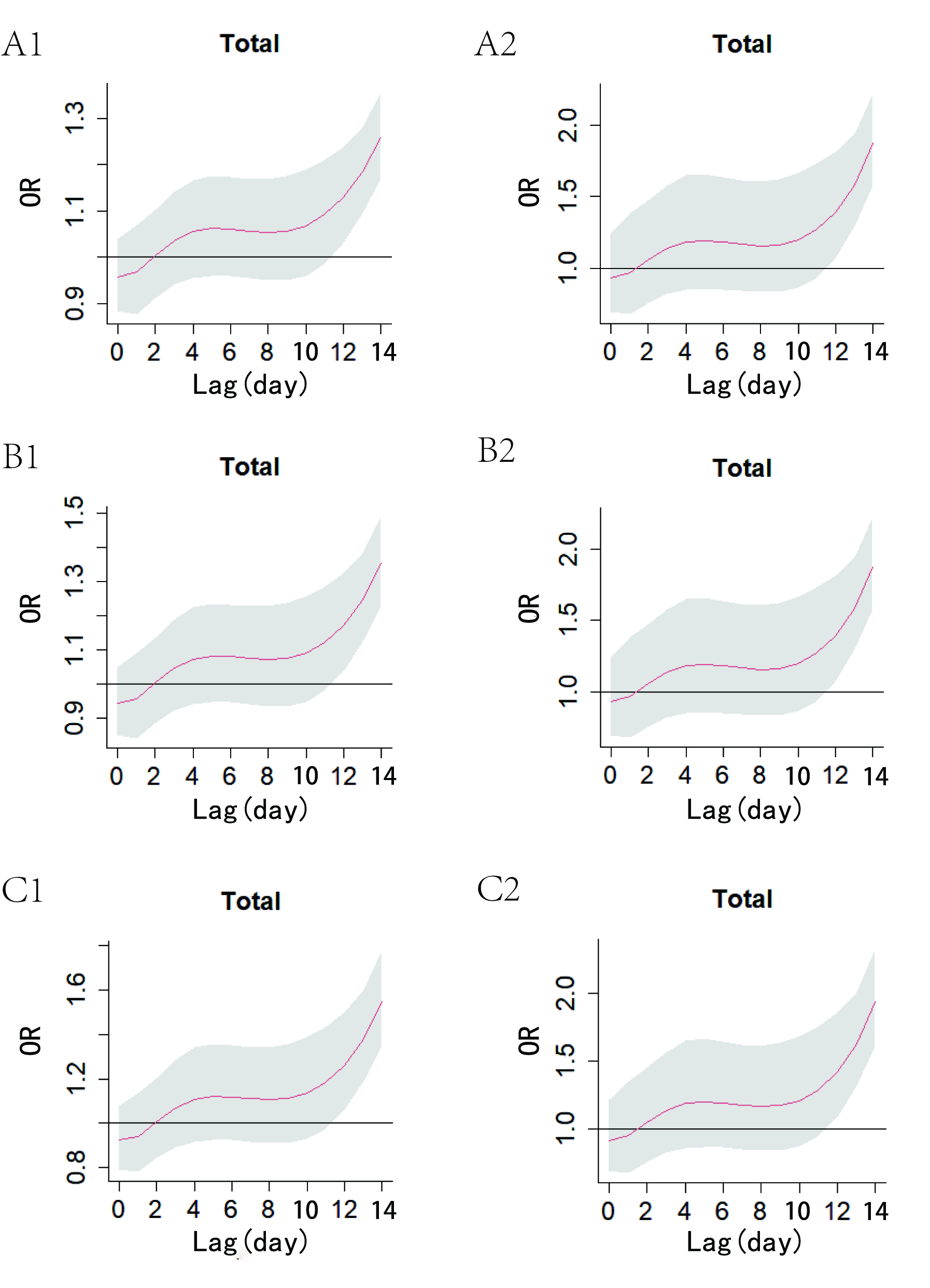


**Figure S2.** Cumulative- Lag- response curves over lag days 0-14 for association between extreme temperature and RTIs fatalities using different temperature threshold values

(A1, A2: P1 & P99; B1, B2: P2.5 & P97.5; C1, C2: P10 & P90.

P1,P2.5,P10: that is the 1st, 2.5th and 10th percentile of temperature distribution as the threshold values of low temperature;

P97.5, P90 , P99: that is the 97.5th, 90th and 99th percentile of temperature distribution as the threshold values of low temperature)

## Supplementary Table

**Table S1** The effects of road traffic injuries when exposed on extreme low and high temperature over lag day 0-14 adjusted by confounders with different degree of freedom

| Confounders | degree of freedom | Extreme low temperature | Extreme high temperature |
| --- | --- | --- | --- |
|  |  | OR (95% CI) | OR (95% CI) |
| RH | 2 | 1.258 (1.168, 1.355) | 1.85 (1.561, 2.192) |
|  | 3# | 1.258 (1.168, 1.355) | 1.85 (1.561, 2.192) |
|  | 4 | 1.258 (1.168, 1.355) | 1.85 (1.561, 2.192) |
| PM2.5 | 2# | 1.258 (1.168, 1.355) | 1.85 (1.561, 2.192) |
|  | 3 | 1.258 (1.168, 1.355) | 1.85 (1.561, 2.192) |
|  | 4 | 1.258 (1.168, 1.355) | 1.85 (1.561, 2.192) |
| PM10 | 2# | 1.258 (1.168, 1.355) | 1.85 (1.561, 2.192) |
|  | 3 | 1.258 (1.168, 1.355) | 1.85 (1.561, 2.192) |
|  | 4 | 1.258 (1.168, 1.355) | 1.85 (1.561, 2.192) |
| NO2 | 2# | 1.258 (1.168, 1.355) | 1.85 (1.561, 2.192) |
|  | 3 | 1.258 (1.168, 1.355) | 1.85 (1.561, 2.192) |
|  | 4 | 1.258 (1.168, 1.355) | 1.85 (1.561, 2.192) |
| SO2 | 2 | 1.258 (1.168, 1.355) | 1.85 (1.561, 2.192) |
|  | 3# | 1.258 (1.168, 1.355) | 1.85 (1.561, 2.192) |
|  | 4 | 1.258 (1.168, 1.355) | 1.85 (1.561, 2.192) |
| CO | 2 | 1.258 (1.168, 1.355) | 1.85 (1.561, 2.192) |
|  | 3# | 1.258 (1.168, 1.355) | 1.85 (1.561, 2.192) |
|  | 4 | 1.258 (1.168, 1.355) | 1.85 (1.561, 2.192) |

Note: Confounders, such as Rh, PM2.5, PM10, NO2, SO2, and Co, were adjusted with different degree of freedom from 2 to 4 in the models. # means the optimal degree of freedom for each confounding factor in the model.
